# Chronotherapy to reinforce circadian rhythms improves poststroke outcomes and glymphatic function in mice

**DOI:** 10.1172/JCI201800

**Published:** 2026-06-15

**Authors:** Emma Waight, Yuxi Zhu, Ashley Caudell, Velia S. Vizcarra, Evan Newbold, Michael J. Giannetto, Evalien Duyvestyn, Estephanie Balbuena, Wei Song, Tanzil M. Arefin, Yuki Mori, Maiken Nedergaard, Lauren M. Hablitz

**Affiliations:** 1 Center for Translational Neuromedicine,; 2 Department of Neuroscience, and; 3 Center for Advanced Brain Imaging and Neurophysiology, University of Rochester Medical Center, Rochester, New York, USA.; 4 Center for Translational Neuromedicine, Faculty of Health and Medical Sciences, University of Copenhagen, Copenhagen, Denmark.

**Keywords:** Cell biology, Neuroscience, Stroke

## Abstract

Stroke remains a leading cause of morbidity and mortality worldwide, with few effective interventions to promote recovery. Targeting circadian timing and glymphatic function may represent viable therapeutic strategies. Here, we show that the small-molecule clock modulator, KL001; high-dose melatonin; acute light pulses; and active-phase time-restricted feeding were each sufficient to enhance glymphatic function in mice. Moreover, initiating treatment with either KL001 or active-phase time-restricted feeding 3 days after preclinical models of stroke improved motor outcomes, reduced lesion volume, increased glymphatic flow, and lowered poststroke brain cytokine burden. These findings suggest that reinforcing normal daily rhythmicity after stroke can markedly enhance neurological recovery, even when interventions are initiated several days after stroke onset.

## Abstract 2

Reinforcing how the body tells time across the day can increase glymphatic flow, reduce brain inflammation, and improve stroke outcomes in preclinical (mouse) models.

## Introduction

Circadian timing regulates 24-hour cycles in almost every aspect of cell biology, physiology, and behavior. These circadian rhythms range from gene transcription and translation to blood pressure and heart rate, to sleep/wake cycles and mealtimes, regulating fundamental homeostatic processes throughout the body (1–5). In the brain, the glymphatic system — a network of perivascular spaces that enables fluid movement and brain waste clearance — is under circadian control (6, 7), with a peak in brain waste clearance during the sleep phase and drainage of cerebrospinal fluid to the lymphatic system during the active phase. Thus, circadian disruption may impair brain waste clearance and homeostasis.

Following stroke, disruption of circadian-regulated motor behavior, including activity during sleep, fragmentation of sleep/wake cycles, and changes in chronotype, have been shown to correlate with the severity of white matter lesions in small vessel disease and severity of ischemic stroke. Moreover, changes in circadian timing negatively correlate with recovery outcomes like poststroke apathy and quality of life, as measured by the SF-36 questionnaire (8–12). One week after stroke there is marked glymphatic impairment both in preclinical models and in stroke patients (13), suggesting that interventions that enhance circadian rhythmicity and glymphatic function may promote stroke recovery.

Here, we test whether chronotherapy — interventions that reinforce the timing of circadian rhythms — can augment glymphatic function and improve stroke recovery. The small molecule KL001 was chosen for the growing interest in manipulating the molecular clock directly (14–16). Melatonin (17–19), light (20–26), and time-restricted feeding (27–29) were chosen because they are already of clinical use in treating diseases that present with circadian disruption. Using preclinical mouse models, we found that with correct timing of administration these interventions regulated glymphatic function. KL001 and active-phase restricted feeding, specifically, improved preclinical models of stroke recovery and reduced cytokine burden in the brain, correlating to improved glymphatic function.

## Results

### Chronotherapy boosts glymphatic function and consolidates 24-hour activity rhythms.

The glymphatic system is regulated by circadian timing (6). Thus, interventions that shift circadian rhythms should also influence glymphatic function. To test this hypothesis, several chronotherapeutic interventions were applied, and glymphatic influx was measured.

**Figure 1:**
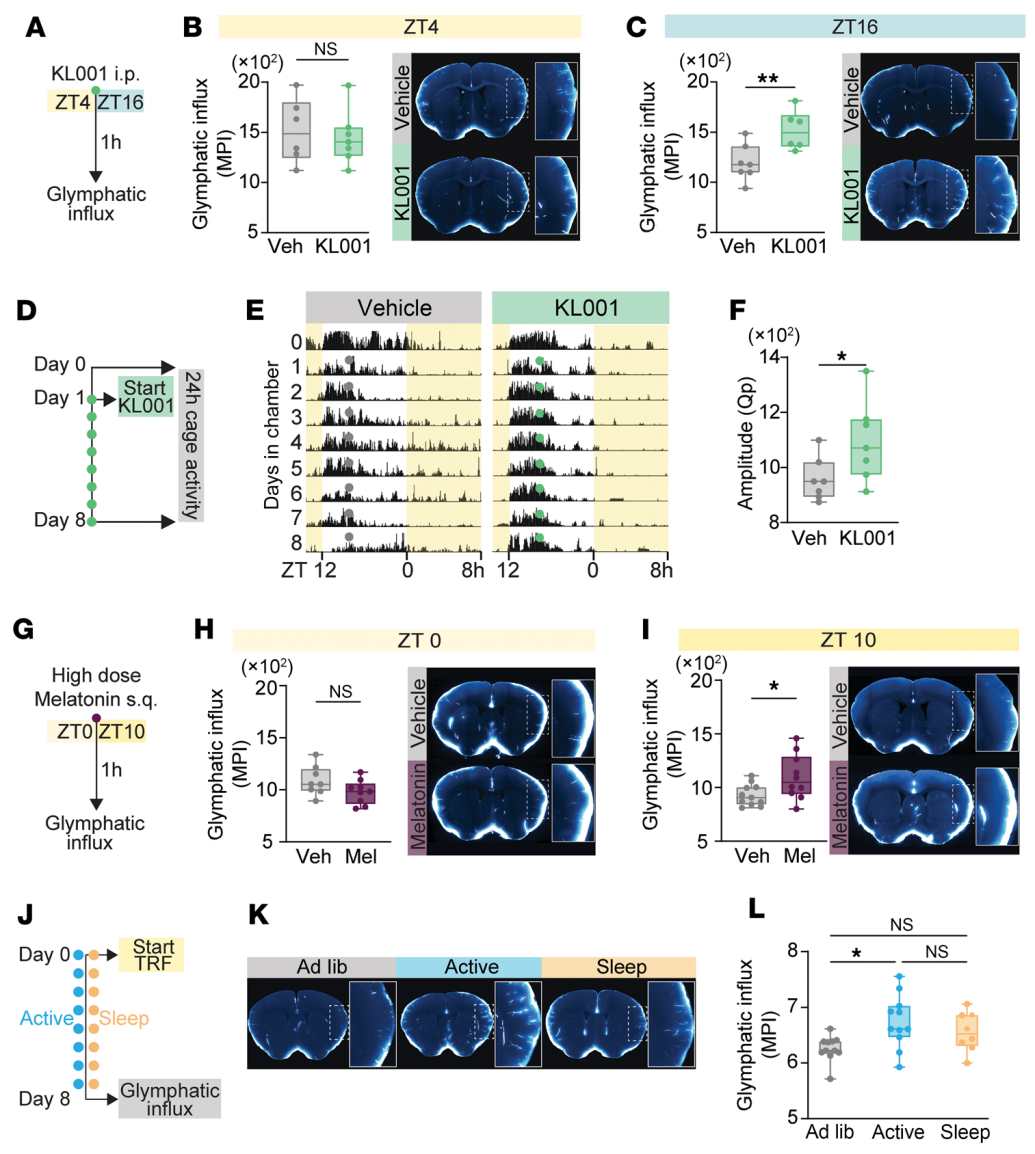
Circadian interventions boost glymphatic flow in healthy mice. (**A**) Experimental design of acute KL001 administration. (**B** and **C**) Box plots of mean pixel intensity (MPI) of glymphatic influx and representative coronal sections for experiments done at Zeitgeber time 4 (ZT4, lights on at ZT0) (**B**) or ZT16 (**C**). (**D**) Experimental design of KL001 treatment and diurnal behavioral monitoring. (**E**) Representative single-plotted actograms of mice receiving vehicle (gray) or KL001 (green) for 8 days. Each treatment is indicated by a colored dot; black tick marks denote general cage activity; white represents lights off; tan indicates lights on. (**F**) Box plot of χ^2^ periodogram calculated rhythmicity amplitude at 24 hours. (**G**) Experimental design of high-dose melatonin administration. (**H** and **I**) Box plots of MPI of glymphatic influx and representative coronal sections for experiments done at ZT0 (**H**) or ZT10 (**I**). (**J**) Experimental design of time-restricted feeding (TRF). (**K**) Representative coronal brain sections for glymphatic influx analysis (left). The region within the dotted box is shown at ×2.5 magnification to the right. (**L**) Box plot of MPI of glymphatic influx. For all box plots, median and quartiles are shown by box-and-whisker plots, with individual mice shown as colored dots. *P < 0.05, **P < 0.01. **B**, **C**, **F**, and **H**: unpaired 2-tailed *t* tests. **I**: unpaired 2-tailed *t* test with Welch’s correction. **L**: 1-way ANOVA test with Tukey’s HSD post hoc comparisons. All statistics are shown in Supplemental Table 1.

Circadian rhythms are controlled on the molecular level by the circadian molecular clock, a transcription-translation negative feedback loop (30). There is a growing interest in developing small-molecule modulators of the molecular clock (15, 16), and these molecules may be useful for regulation of glymphatic function. First, we tested KL001, a small-molecule modulator of the circadian clock that stabilizes cryptochrome (14). Cryptochrome expression reaches a nadir at zeitgeber time 4 (ZT4, where ZT0 marks lights on) and peaks at ZT16 in the cortex (31), a key region for glymphatic influx. KL001 was administered at ZT4 or ZT16 (Figure 1, A–C). No effect was observed at ZT4, but KL001 given at ZT16 significantly enhanced glymphatic influx. Moreover, repeated daily KL001 treatment for 8 days at ZT16 increased the amplitude of 24-hour cage activity rhythms compared with that in vehicle controls (Figure 1, D–F). These findings demonstrate that KL001 both strengthens daily rhythmicity and enhances glymphatic function in healthy wild-type mice.

Most small-molecule modulators of the molecular clock are still in development and are currently not suitable for clinical care. However, there are circadian interventions already in use in the clinic. Melatonin, widely used to treat circadian rhythm sleep disorders, was also tested (17–19). Melatonin has the greatest phase-shifting effect on circadian timing at ZT10, with minimal phase shifts at ZT0 in both mice and humans (17, 18, 32, 33). When administered at ZT10, melatonin significantly increased glymphatic function (Figure 1, G–I), but no effect was observed at ZT0. However, at a lower clinically relevant dose (32), melatonin failed to enhance glymphatic function (Supplemental Figure 1, A and B; supplemental material available online with this article; https://doi.org/10.1172/JCI201800DS1). Treatment with melatonin for 2 weeks at ZT10 at the higher dose failed to significantly change glymphatic influx (Supplemental Figure 1C), suggesting that other circadian-targeted interventions may be more suitable for increasing glymphatic function.

Bright-light therapy has been used clinically for depression, epilepsy, ADHD, and more (20–26). Light is the most potent circadian time (CT) cue. In mammals, light pulses during constant dark can shift behavioral rhythms up to 4 hours (34–36). To test whether light affects glymphatic function, mice housed in constant darkness for 9 days were exposed to 15 minutes of either white light or red light at the beginning of the active phase (CT14, where CT12 marks activity onset). Glymphatic influx was significantly increased under white light compared with that under red light at CT14 (Supplemental Figure 1, C and D; Supplemental Table 2). In contrast, no effect was observed when white light was administered at the beginning of the sleep phase, CT2, compared with red light controls (Supplemental Figure 1D).

Finally, time-restricted feeding is an established, noninvasive, and nonpharmacological intervention for improving circadian timing in cardiometabolic disease (27–29). Restricted feeding to the active phase significantly increased glymphatic influx compared with ad libitum feeding, whereas restricted feeding to the sleep phase had no effect (Figure 1, J–L). These data suggest that circadian interventions, including KL001, high-dose melatonin, light, and active-phase restricted feeding, may represent viable interventions for disorders involving circadian and glymphatic dysfunction.

### Stroke impairs 24-hour behavioral rhythms and glymphatic function.

**Figure 2:**
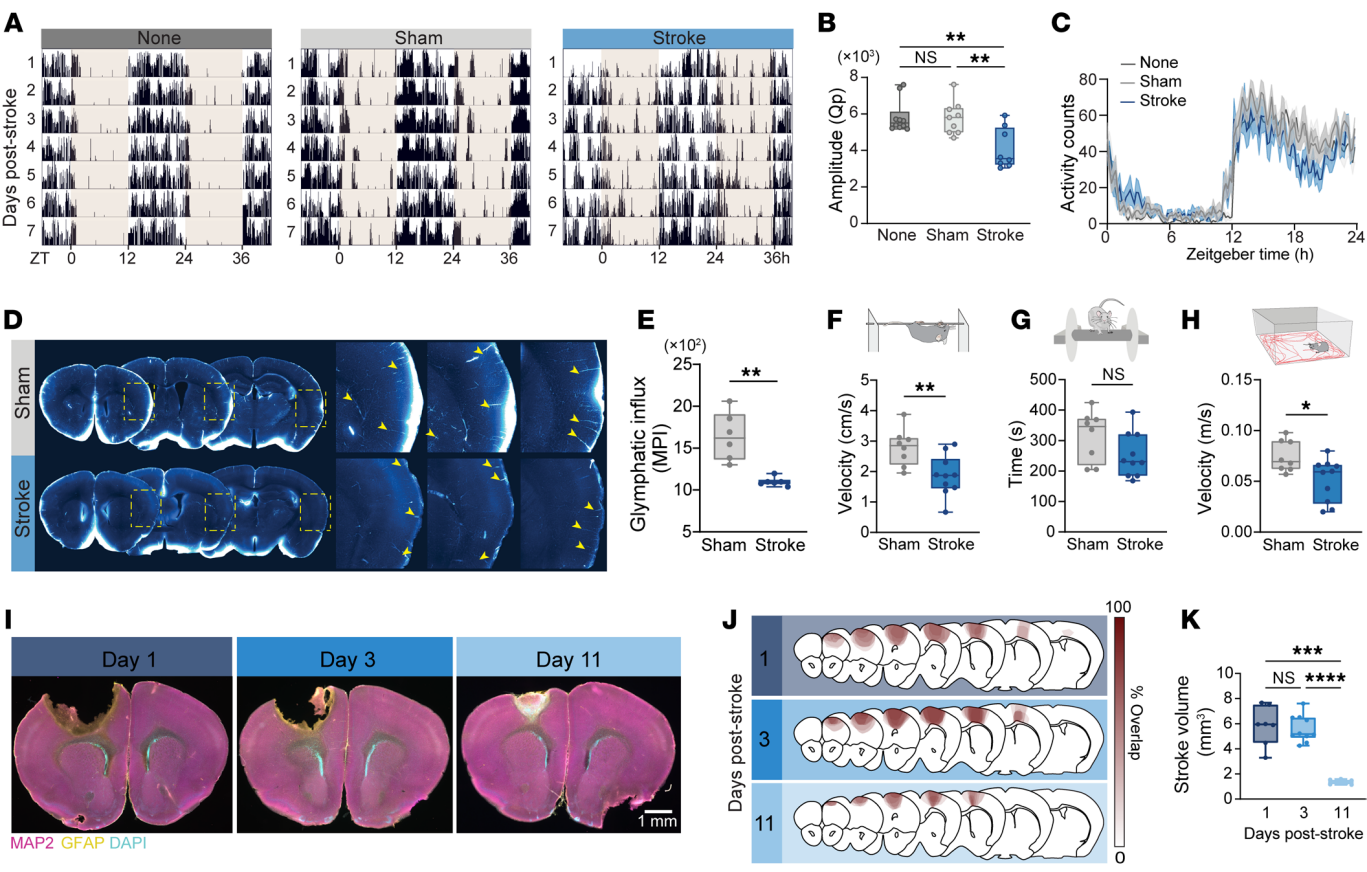
Ischemic stroke disrupts circadian behavior and glymphatic flow. (**A**) Representative double-plotted actograms of double-housed mice that received no surgery (None, dark gray), sham surgery (light gray), or photothrombotic stroke (blue). Tan indicates lights on; white represents lights off; black tic marks denote wheel-running activity. (**B**) Box plot of χ^2^ periodogram calculated rhythmicity amplitude at 24 hours. (**C**) Average activity counts after stroke over 24 hours. Thick lines indicate means, and shading indicates SEM. (**D**) Representative coronal sections of glymphatic tracer influx. Lighter colors/white are increased fluorescent tracer. Area within yellow boxes is shown at ×3 magnification to the right, with yellow arrows indicating perivascular influx of CSF tracer. (**E**) Box plot of MPI of glymphatic influx after sham and stroke. (**F**–**H**) Cartoon representative of the behavior tests: box plots of wire walk (**F**), rotarod (**G**), and velocity in open field (**H**) for sham (gray) and stroke (blue) mice. (**I**) Representative coronal immunohistochemistry (IHC) sections for mice 1 day, 3 days, and 11 days after photothrombotic stroke. MAP2 (magenta) was used to identify the stroke site. GFAP (yellow) and DAPI (cyan) were used for slice visualization. Scale bar: 1 mm. (**J**) Schematics of anterior-to-posterior coronal sections (white) with individual mouse stroke sites indicated in transparent red at 1, 3, and 11 days after stroke. The darker the red, the more stroke sites overlapped between mice. (**K**) Minimum/maximum box plot of stroke volume in mice 1, 3, and 11 days after stroke. In all box plots, minimum and maximum values are shown, center lines indicate the medians, quartiles are shown by box-and-whisker plots, individual mice are shown as colored dots. *P < 0.05, **P < 0.01, ***P < 0.001, ****P < 0.0001. **B**: 1-way ANOVA test with Tukey’s HSD post hoc analysis. **F**–**H**: unpaired *t* tests. **E**: unpaired *t* test with Welch’s correction. **K**: Welch’s ANOVA test with Dunnett’s T3 multiple comparisons test. All statistics are shown in Supplemental Table 1.

We next tested the hypothesis that circadian behavioral disruption occurs in preclinical models of stroke. Photothrombotic strokes were induced via photoactivation of the dye rose bengal and compared with sham-operated and nonsurgery control mice (Figure 2, A–C). Using χ^2^ periodogram analysis over days 1–7 after stroke, photothrombotic stroke injury significantly reduced the amplitude of 24-hour general cage activity relative to both sham-operated mice and nonsurgery controls (Figure 2, A and B). No differences were observed between mice that did not undergo surgery and sham controls. Importantly, there were no significant changes in average daily activity, dark activity, or light activity across all groups, demonstrating that the reduction in rhythmicity was not driven by impaired wheel running (Figure 2C and Supplemental Figure 2, A–C).

These behavioral changes may reflect altered brain homeostasis, such as perivascular flow dynamics within the glymphatic system. Consistent with this, mice exhibited reduced glymphatic influx (Figure 2, D and E). Functionally, poststroke mice showed impaired performance on the wire walk test (Figure 2F) and reduced total distance traveled and average speed in the open-field test (Figure 2H and Supplemental Figure 2, D–G) compared with sham controls. No group differences were detected on the rotarod test (Figure 2G). Together, these findings demonstrate that daily rhythms are disrupted after stroke and may represent a viable therapeutic target. Reinforcing the intrinsic timing of physiological processes such as glymphatic clearance could improve the removal of cellular debris from the injured brain and promote recovery.

To better understand the natural course of recovery, we compared lesion morphology at 1, 3, and 11 days after stroke (Figure 2, I–K). Lesion volume remained unchanged between days 1 and 3 but was significantly reduced by day 11. These findings suggest that chronotherapeutic interventions initiated during the subacute phase after photothrombotic stroke, specifically days 3–11, may be both feasible and effective.

### KL001 chronotherapy improves recovery in permanent ischemia.

**Figure 3:**
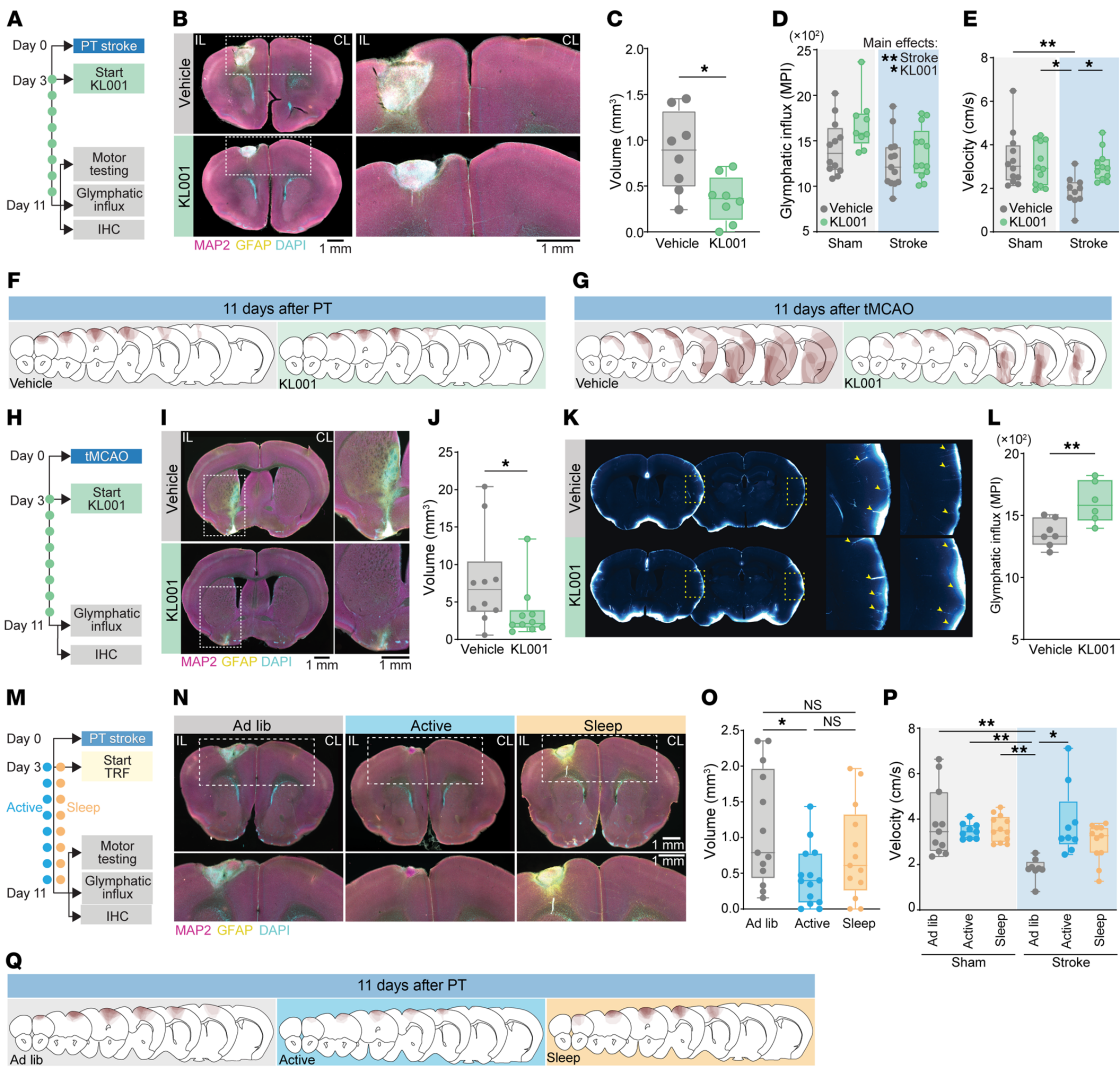
Chronotherapy improves glymphatic function and stroke recovery. (**A**) Schematic of experiment for KL001 treatment after photothrombotic (PT) stroke. (**B**) Representative IHC (scale bar: 1 mm) for box plot of (**C**) stroke volume, (**D**) MPI of glymphatic influx, and (**E**) wire walk velocity. (**F**) Schematics of anterior-to-posterior coronal sections (white) with individual mouse photothrombotic stroke sites indicated in transparent red for both vehicle (gray) and KL001 (green). The darker the red, the more stroke sites overlapped between mice. (**G**) Same as in **F**, but for transient middle cerebral artery occlusion–induced (tMCAO-induced) lesions. (**H**) Schematic of experiment for KL001 treatment after tMCAO. (**I**) Representative IHC (scale bar: 1 mm) for (**J**) box plot of stroke volume. (**K**) Representative coronal sections of fluorescent tracer after glymphatic influx assay. Areas within dotted yellow boxes are shown at ×2.5 magnification to the right, with perivascular tracer penetration highlighted with yellow arrows. Images are quantified by the box plot in **L**. (**M**) Schematic of experiment for restricted feeding after photothrombotic (PT) stroke. (**N**) Representative IHC (scale bar: 1 mm) for box plot of (**O**) stroke volume and (**P**) wire walk velocity. (**Q**) Same as in **F**, but for photothrombotic lesions after restricted feeding. Ad libitum (gray), active fed (blue), and sleep fed (orange) groups are indicated by shading. For all IHC, MAP2 (magenta), GFAP (yellow), and DAPI (cyan) 11 days after stroke. IL, ipsilateral; CL, contralateral. Area with dotted white boxes is shown at higher magnification below. Gray shading indicates sham, and blue shading indicates stroke. For all box plots: vehicle (gray), KL001 (green), active feeding (light blue), sleep feeding (orange) mice. Median and quartiles are shown by box-and-whisker plots, with individual mice shown as colored dots. *P < 0.05, **P < 0.01. **C** and **L**: unpaired *t* tests. **D** and **E**: 2-way ANOVA with Tukey’s HSD post hoc analysis. **J**: Mann-Whitney test. **O**: 1-way ANOVA test with Tukey’s HSD post hoc analysis. **P**: Kruskal Wallis test and Dunn’s multiple comparisons test. All statistics are shown in Supplemental Table 1.

We next tested whether repeated treatment with KL001 enhances stroke recovery. Beginning 3 days after induction of photothrombotic strokes, when the lesion was established, KL001 or vehicle was administered at ZT16 for 8 consecutive days (Figure 3, A–F). KL001 treatment significantly reduced stroke volume compared with that in vehicle controls (Figure 3, B, C, and F). As expected, glymphatic influx was suppressed in mice in the stroke group, but KL001 increased influx in both sham and stroke groups (Figure 3D).

In parallel, KL001 improved circadian outputs, consolidating the activity length by approximately 20 minutes (Supplemental Figure 3, A–D) without affecting the total number of activity counts or activity distribution across day and night (Supplemental Figure 3). Functionally, KL001 significantly increased velocity on the wire walk test after stroke (Figure 3E). These findings indicate that KL001 treatment initiated 3 days after stroke reduces stroke lesion size, restores glymphatic function, improves 24-hour timing of behavioral rhythms, and enhances motor recovery.

### KL001 also improves recovery after transient ischemia.

Can KL001 treatment also enhance recovery after the transient middle cerebral artery occlusion (tMCAO)? To answer this question, mice received KL001 or vehicle for 8 consecutive days beginning 3 days after tMCAO. KL001 treatment significantly reduced lesion size and increased glymphatic influx compared with that in vehicle controls (Figure 3, G–L). These findings support that KL001 therapy may represent a novel approach to improve stroke recovery, independent of the ischemia model.

### Active-phase–restricted feeding increases glymphatic function and improves stroke recovery.

We next tested whether time-restricted feeding, a behavioral chronotherapy, could improve recovery after stroke. The central hypothesis was that reinforcing endogenous circadian timing enhances poststroke recovery processes and that interventions providing sufficiently strong time cues would have effects similar to KL001. Three days after photothrombosis, mice were assigned to active-phase–restricted feeding, sleep-phase–restricted feeding, or ad libitum feeding for 8 days (Figure 3, M–Q). Compared with ad libitum controls, active-phase feeding increased glymphatic influx (Supplemental Figure 4, A, B, and D), although this effect did not remain significant when sleep-fed mice were included in the statistical model (Supplemental Figure 4, A–C). Importantly, sleep-phase feeding did not increase glymphatic function regardless of model (Supplemental Figure 4, C–E), suggesting that mistimed food may not impair glymphatic function. Active-phase feeding also reduced stroke volume (Figure 3, N, O, and Q) and improved performance on the wire walk test (Figure 3P). In contrast, sleep-phase feeding had no effect on glymphatic influx, stroke volume, or motor outcomes.

These results indicate that restricting feeding to the active phase improves glymphatic function, reduces ischemic damage, and enhances motor recovery, supporting the conclusion that reinforcing circadian timing through either pharmacological or behavioral interventions can promote recovery after ischemic injury.

### Longitudinal assessment confirms the benefit of chronotherapy in stroke recovery.

**Figure 4:**
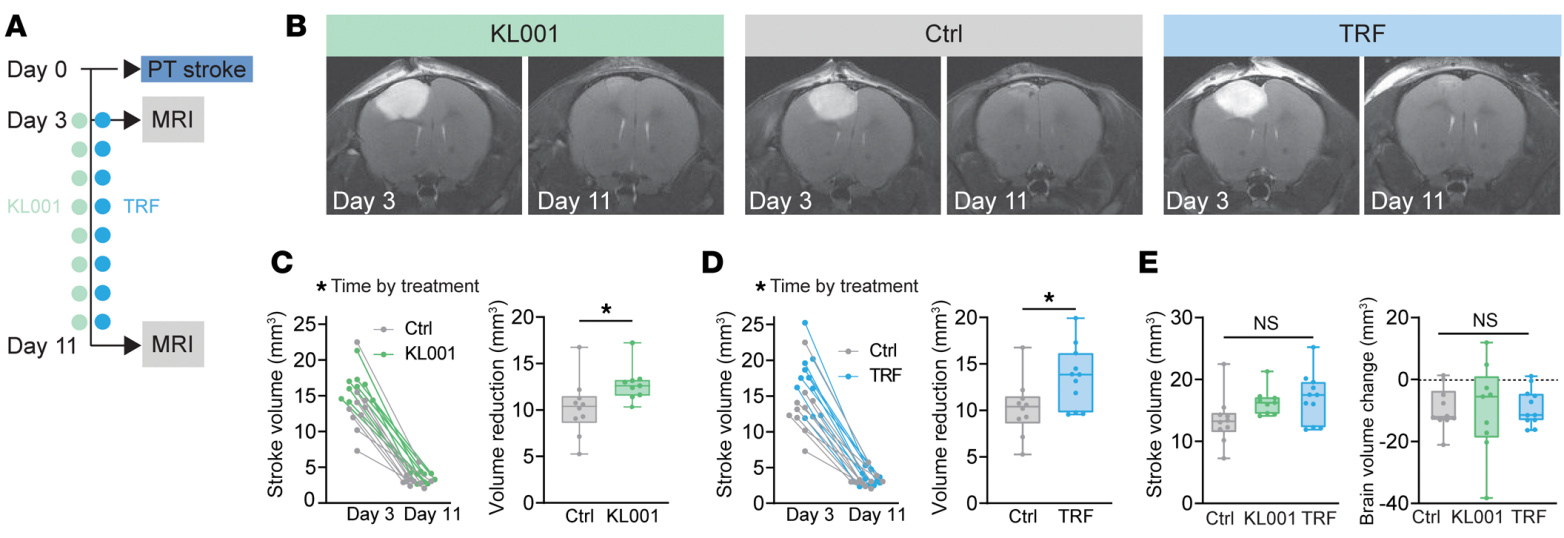
Chronotherapy improves stroke outcome in longitudinal MRI. (**A**) Experimental design. (**B**) Representative coronal images of T_2_-weighted MRI before and after chronotherapy with KL001 (green), control (Ctrl, gray), or active-phase restricted feeding (TRF, blue) that began on day 3 after stroke. The image of the first section anterior to where corpus callosum was continuous was selected. (**C**) (left) Paired scatterplot of stroke volume for mice day 3 before chronotherapy and day 11 after chronotherapy with KL001. (right) Box plot of total volume reduction in lesion size before minus after chronotherapy. (**D**) Same as in **C**, but with active-phase restricted feeding (blue). (**E**) (left) Box plot of stroke volume on day 3, before chronotherapy. (right) Box plot of total brain volume change (day 11 minus day 3) for control- (gray), KL001- (green), and TRF-treated (blue) groups. For all box plots, vehicle (gray), KL001 (green), active feeding (light blue) mice. Median and quartiles are shown by box-and-whisker plots, with individual mice shown as colored dots. *P < 0.05. **C**, left, and **D**, left: 2-way ANOVA tests with repeated measures. **C**, right: Mann-Whitney test. **D**, right: unpaired 2-tailed *t* test. **E**, left: 1-way ANOVA test. **E**, right: Welch’s ANOVA. All statistics are shown in Supplemental Table 1.

Although photothrombotic lesions at days 3 and 11 were consistent, reproducible, and quantifiable (Figure 2, I–K), it would be ideal to analyze lesion volume at both time points (days 3 and 11 after injury) in the same mice. To accomplish this, mice underwent photothrombosis, and T_2_-weighted MRI was used to assess lesion volumes at day 3 and day 11 after stroke. Between imaging time points, mice were maintained in control conditions (ad libitum food), treated with KL001 for 8 days, or subjected to active-phase–restricted feeding for 8 days (Figure 4A).

As expected, lesions measured via MRI (Figure 4, B–D) were larger than those measured postmortem at days 3 and 11 after stroke (Figures 2 and 3). This is because in vivo tissue has not been exposed to paraformaldehyde fixation, where the osmolality of the 4% paraformaldehyde solution is >4-fold higher than most biological fluids and tissues, which drives water loss and shrinkage of biological samples (37, 38).

At day 3, stroke volumes were similar across groups (Figure 4E, left). Between days 3 and 11, both KL001 treatment and active-phase feeding significantly reduced lesion volume relative to that in controls (Figure 4, C and D). Importantly, this reduction was not attributable to tissue loss, as no differences in total brain volume were observed across groups before or after treatment (Figure 4E, right). These longitudinal findings confirm that chronotherapy, whether pharmacological or behavioral, supports improved recovery after stroke.

### Chronotherapy selectively reduces brain cytokine burden after stroke.

**Figure 5:**
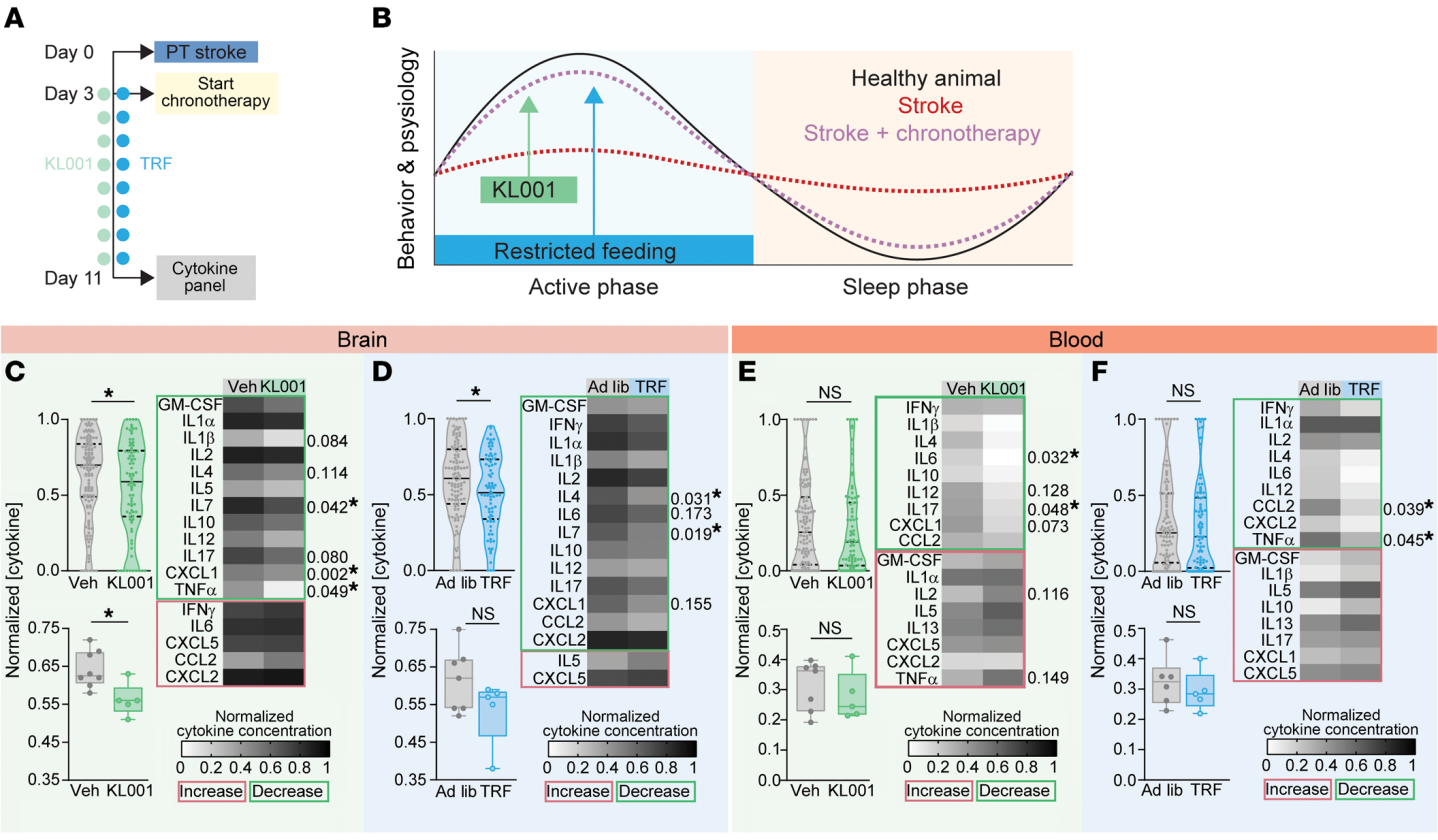
Chronotherapy reduces brain, but not blood, cytokine burden after stroke. (**A**) Schematic of experimental design. (**B**) Model figure. Daily cycles in behavior and physiology (black line) are dampened in stroke (dotted red line), leading to decreased glymphatic function and cytokine accumulation. Chronotherapy (KL001, green; active-phase restricted feeding, blue) can reinforce 24-hour behavior and physiology (purple line). Glymphatic function is increased, cytokine burden is decreased, and stroke recovery is improved. An important note is that although chronotherapy may reinforce timing, the change in endogenous rhythms may be intervention and disease specific. (**C**) Violin plot of all individual brain cytokine concentrations normalized to the peak value with and without KL001. Box plot of normalized cytokine concentration by mouse. Normalized group mean of cytokine concentrations. Darker gray indicates increased concentration, green border indicates decreases with KL001, and red border indicates increases with KL001. Values to the right are all *P* values under 0.2 for group comparisons. (**D**) Same as in **C**, but with active-phase time-restricted feeding. (**E**) Same as in **C** and **D**, but measured in blood after KL001 chronotherapy. (**F**) Same as in **E**, but measured in blood after active-phase time-restricted feeding chronotherapy. For violin plots, vehicle/ad libitum (gray), KL001 (green), and active-phase time-restricted feeding (blue). Width reflects concentration of data, dotted lines indicate upper and lower quartile limits, solid black lines indicate median, and individual points represent individual cytokine values. For box plots, vehicle/ad libitum (gray), KL001 (green), and active-phase time-restricted feeding (blue). Minimum and maximum values are shown, center lines indicate the medians, and quartiles are shown by box-and-whisker plots, with individual mice shown as colored dots. *P < 0.05, **P < 0.01, ***P < 0.001. An unpaired *t* test, an unpaired *t* test with Welch’s correction, or a Mann-Whitney test were used for statistical tests across panels. All statistics are shown in Supplemental Table 1.

KL001 and active-phase–restricted feeding improve stroke recovery, along with enhancing glymphatic function. Yet, how increased glymphatic function may improve stroke recovery remains unclear. Stroke triggers neuroinflammation, releasing cytokines from injured tissue, initiating a peripheral immune response (39). We hypothesized that chronotherapy increases glymphatic function, ultimately reducing cytokine burden in the brain, contributing to improved recovery. To test this, KL001 or active-phase–restricted feeding was applied for 8 days, beginning 3 days after photothrombotic stroke. Cytokine levels 11 days after stroke were then quantified in brain homogenate and blood plasma using a multiplex panel (Figure 5, A–F and Supplemental Figure 5).

In the brain, cytokine levels decreased after both KL001 and active-phase–restricted feeding (Figure 5, C and D). This reduction reached statistical significance in individual mice treated with KL001 (Figure 5C, bottom) but was a trend in the active-phase feeding group (*P* = 0.1423, Figure 5D, bottom, and Supplemental Table 1). Importantly, an analysis of control photothrombotic stroke brains resulted in no significant differences in cytokine burden between contra- and ipsilateral sides to the lesion 11 days after stroke (Supplemental Figure 6), demonstrating that this bulk change in cytokine levels was likely not due to inclusion of the stroke lesion in the sample. Of the 17 cytokines measured, only IL-12 was upregulated in the contralateral side to the stroke (Supplemental Figure 6C), supporting this conclusion.

Among the 17 cytokines measured in the KL001 group, 12 showed reduced mean levels relative to those of vehicle controls. Three cytokines (IL-7, CXCL1, and TNF-α) were significantly decreased, while 3 others (IL-1β, IL-4, and IL-17) showed trends toward decreasing. A similar pattern was observed with active-phase feeding: 14 of 16 cytokines measured displayed decreased mean levels (Figure 5C, right, and Figure 5D, right). In contrast, cytokine changes in blood plasma were less pronounced (Figure 5, E and F). Only 9 of 17 cytokines (53%) decreased after chronotherapy, compared with 71% and 88% of cytokines in the KL001 and active-phase brain groups, respectively.

In summary, both pharmacological (KL001) and behavioral (active-phase feeding) chronotherapy reduced global cytokine levels in brain after stroke, which correlated with enhanced glymphatic function. These findings support a mechanism by which circadian reinforcement alleviates neuroinflammation and promotes recovery after stroke (Figure 5B).

## Discussion

Restricted feeding paradigms have gained traction as interventions for multiple diseases, including obesity (40), heart disease (41–44), and Alzheimer’s disease (45). In the context of stroke, time-restricted feeding has previously been tested as a preventative lifestyle intervention (46–48). Our study is unique, demonstrating that time-restricted feeding can be used therapeutically after preclinical models of stroke to promote recovery, rather than a solely prophylactic measure. Our analysis showed that both time-restricted feeding and pharmacological targeting of circadian timing with KL001 improved stroke recovery in preclinical models. By defining efficacious parameters and temporal windows of behavioral and pharmacologic chronotherapy, circadian rhythms can be reinforced to improve stroke outcome. More broadly, interventions targeted for management of the circadian system may improve outcomes in disorders where time-of-day effects contribute to pathophysiology.

The most effective stroke treatments currently focus on the rapid restoration of blood flow through clot dissolution or removal within hours of onset (49–54). Tissue plasminogen activator therapy and mechanical thrombectomy are most beneficial when administered within 4 and 16 hours of symptom onset, respectively (55, 56); however, fewer than 5% of patients meet these strict treatment criteria (57, 58). A long list of neuroprotective agents developed experimentally have failed in clinical trials, largely because their efficacy is limited to administration within hours of stroke onset (59, 60). Poststroke rehabilitation remains the mainstay of long-term care, but its availability and quality vary widely depending upon the degree of disability, socioeconomic resources, and geographic location (61, 62). Notably, up to 1 in 3 patients may lack access to adequate clinic-to-home rehabilitation (54), underscoring the urgent need for accessible, long-term interventions to improve recovery and quality of life. Here, we show that chronotherapeutic interventions initiated several days after ischemic injury can mitigate morbidities associated with delayed stroke treatment. Circadian-based interventions such as timed feeding or bright-light exposure represent low-cost, low-maintenance lifestyle adjustments that could be readily implemented to enhance recovery outcomes in healthcare settings and at home.

Following ischemic stroke, cerebral edema evolves in a predictable temporal pattern. Cytotoxic edema develops rapidly within hours of arterial occlusion, followed by vasogenic edema driven by blood-brain barrier disruption and inflammatory responses (63, 64). In most rodent and human studies, total ischemic edema volume peaks between poststroke days 2 and 4. During this window, tissue swelling reflects intracellular water accumulation, extracellular fluid expansion, and impaired fluid clearance. Thereafter, edema gradually resolves over days to weeks as excess interstitial and intracellular fluid is resorbed through vascular, lymphatic, and glymphatic pathways, and as blood-brain barrier integrity is partially restored (65–67). As edema subsides, the apparent size of the ischemic lesion on imaging correspondingly decreases, not necessarily because viable tissue is recovered, but because the transient water component diminishes, and the infarct matures into a more compact area of tissue loss and gliosis.

An additional methodological consideration is that histological processing alters tissue dimensions. Fixation with 4% paraformaldehyde, which is hyperosmotic relative to brain tissue, induces osmotic water efflux and protein crosslinking, leading to shrinkage. Reported linear shrinkage of rodent brain tissue after fixation in 4% paraformaldehyde typically ranges from approximately 5% to 15%, depending on fixation conditions (37, 38). These effects must be considered when comparing in vivo imaging with postmortem histology (Figures 2–4), as both edema resolution and fixation-induced shrinkage reduce the apparent infarct size.

Both interventions tested here — active-phase administration of KL001 and active-phase time-restricted feeding — are correlated to improved stroke recovery and increased glymphatic function. Glymphatic decline has been described in normal aging (68), neurodegenerative disease (69), and across a broad spectrum of neurological disorders (70). Though this study is largely correlational, the findings of an interplay between circadian timing and glymphatic function point to the conclusion that improving brain fluid transport, in addition to circadian timing, represents a promising axis for therapeutic intervention. For example, future studies may determine whether chronotherapy like KL001 is effective because it increases the baseline of known circadian rhythms in glymphatic function (6) or because it is applied when glymphatic function is most susceptible to manipulations of the molecular clock in the cortex. Understanding how circadian regulation shapes glymphatic clearance will enable the development of targeted chronotherapies designed to optimize brain waste clearance, reduce neuroinflammation, and improve recovery after neurological injury.

## Methods

### Sex as a biological variable.

All groups included both male and female mice. No sex-dependent effects were found in these datasets.

### Animals.

Male and female wild-type, C57BL/6NCrl mice (aged 2–4 months, weight of between 25 and 30 g) were used for these experiments. Similar findings are reported for both sexes. These mice were bred in the University of Rochester vivarium, with breeders refreshed from Charles River Laboratories every generation. Although C57BL/6 mice are melatonin deficient, studies have confirmed that melatonin binding, receptor localization, and circadian phase-shifting effects of melatonin are still intact and comparable to other melatonin-competent mouse strains (17, 18, 71–73). Mice were group housed in a 12-hour light and 12-hour dark cycle, with ad libitum access to food and water, except for during the time-restricted feeding experiments, for which food was withheld for 12 hours during either the light or dark phase of the animal for 8 days starting on day 3 after surgery. All mice were given at least 1 week to acclimate to cages prior to any surgery or intervention. All of the University of Rochester’s animal holding rooms are maintained within temperature (18°C–26°C) and humidity ranges (30%–70%) described in the Institute for Laboratory Animal Research Guide for the Care and Use of Laboratory Animals (1996). All efforts were made to keep animal usage to a minimum. All experiments were approved by the University of Rochester Medical Center Committee on Animal Resources. A minimum of 5 mice were used in each group. All experiments were done in at least 2 separate experimental cohorts to ensure reproducibility of phenotypes. All exact mouse numbers and statistics are stated in Supplemental Tables 1 and 2.

### Photothrombosis.

Photothrombosis was given in the late sleep phase (ZT8–ZT12). All strokes were centralized over motor cortex (mean ± SEM, 1.68 ± 0.06 mm anterior to bregma) (74–77). Mice were anesthetized with 1.5%–2% continuous inhaled isoflurane. Anesthesia depth was verified by the pedal reflex test. Anesthetized mice were fixed in a stereotaxic frame, the dorsal surface of the skull was exposed, and a dental drill was used to drill a shallow circular divot in the skull. Any surgery that resulted in the drill going all the way through the skull was excluded from the cohort. The mouse was removed from the frame and treated i.p. with 110 mg/kg rose bengal (Sigma-Aldrich) in PBS. The rose bengal was given at least 5 minutes prior to a 532 nm wavelength laser (PSU-H-LED and 1.25 mm fiber cannula; INPUT, 100~240VAC 47~63 Hz 5A; OUTPUT, 140 mW, Changchun New Industries, Optoelectronics Tech. Co. LTD) directed via fiber optic thread to the center of the divot for 15 minutes. After laser exposure, the surgical opening was closed with 5.0 nylon surgical sutures (Med Vet International). Antibiotic ointment (Polyamide, Med Vet International) was applied directly to the surgery site, and local analgesia, 0.25% bupivacaine HCl (Eugia Us), was given subcutaneously at the site of suturing before removing the mice from anesthesia. Mice rested on rodent heating pads throughout surgery, recovery to maintain body temperatures, and then returned to home cages.

### tMCAO.

All tMCAO inductions occurred between ZT6 and ZT12. The longer time window was necessary due to the complexity of the surgery. Mice were anesthetized with 1.5%–2% continuous inhaled isoflurane. The right MCA was occluded with 86% ± 6% (mean ± SEM) reduction in blood flow values for 40 minutes using a 7-0 nylon monofilament (Doccol Corporation) inserted via the external carotid artery through to the internal carotid artery. Cortical blood flow was continuously monitored by Laser Doppler flowmetry (Perimed). There was no difference in percentage reduction of blood flow between groups. The caliber of the filament was chosen in accordance with the recommendation from the supplier’s website depending on the animals’ weight. Rectal temperature was maintained at 37°C ± 0.5°C using a water heating system (Gaymar). After occlusion, the inserted filament was removed, and MCA blood flow was restored, as confirmed by flowmetry. After suturing the surgical wounds, mice were transferred to cages for postsurgical observation. Mice were given 0.125 mL carprofen (5 mg/kg in saline, Vet one) i.p. and 0.5 mL saline (0.9% in water, Ricca) i.p. every 24 hours for 4 days and housed with food and water ad libitum. This protocol was established over the course of 2 pilot experiments, both of which displayed the same trend for the KL001 effect but were statistically underpowered.

### Drugs.

Anesthesia for glymphatic influx experiments was i.p. 100 mg/kg racemic ketamine (Hikma) and 20 mg/kg xylazine (Anased Injection) i.p. (KX). Depth of anesthesia was determined by the pedal reflex test; if the mouse responded to toe pinch, an additional one-tenth the initial dosage was given, and the tracer experiment delayed until full unconsciousness was obtained. Directly prior to cisterna magna (CM) infusion, the animal received an additional one-tenth the initial dosage, and the pedal reflex was tested every 5–10 minutes during the tracer circulation time to ensure proper anesthesia throughout the study. Note, previous studies have shown that KX anesthesia does not affect the timing of glymphatic function (6). KL001 (Tocris Bioscience) was dissolved in a small volume of dimethyl sulfoxide (DMSO, Sigma-Aldrich) and diluted with sterile saline at a dose of 5 mg/kg, about 200 μL per mouse, given i.p. Control animals were injected with saline containing the equivalent volume of DMSO (3% DMSO/saline) in place of the dissolved compound. For melatonin, mice were treated with vehicle (<3% ethanol/saline, s.c.) or melatonin (high dose: 90 μg/mouse, low dose: 1.67 μg/mouse), as described previously (18). All batches of melatonin were validated in-house using the University of Rochester Medical Center Mass Spectrometry Resource Lab.

### 24-hour behavior analyses.

Animals were housed 2 per cage to reduce risk of hypothermia. Previous work has demonstrated this does not impede behavioral analysis (6). For KL001 behavioral experiments, activity was monitored continuously in 5-minute bins via the Comprehensive Lab Animal Monitoring System (Columbus Instruments). Circadian behavioral analysis was completed with ActogramJ. A ***χ***^2^ periodogram analysis was used to confirm behavioral rhythmicity, and average activity onset and offset was calculated for each animal (78) over the course of the treatment. Activity length was calculated as activity offset minus activity onset. For experiments measuring the impact of sham surgery and stroke on rhythmicity, as well as experiments applying white light, wheel-running activity was recorded continuously in 5-minute bins and analyzed using Clocklab software (Actimetrics). Rhythmicity of animal pairs was measured by χ^2^ periodogram analysis. For constant dark experiments, active and inactive phases were calculated using Clocklab software, and mice were removed for CM injections at CT2 or CT14. Each surgery took approximately 15 minutes and was done in a room with either red or white light. Light is signaled to the circadian system by intrinsically photosensitive melanopsin-containing retinal ganglion cells that respond most reliably to blue light (79, 80). Thus, white light has a larger effect than red light on the circadian system, and red light is used as a control for light exposure overall. Mice returned to darkness after the infusion was done and euthanized in darkness at the end of tracer circulation (described below).

### Open-field test.

The open-field arena (60 × 45 × 40 cm^3^, white background) was in a soundproof room with an indirect artificial light source. Under pads of the arena were changed between each mouse to minimize odor cues. Mice from different groups were tested in randomized order throughout the trials. One mouse at a time was placed in the center of the arena, and spontaneous behavior was recorded for 8 minutes (High-Definition Video Camera, Microsoft lifeCam Cinema). After the trials, mice returned to their home cage. Videos were evaluated using ANY-maze video tracking system (Stoelting Co.), which measured distance moved (cm), velocity (m/s), time mobile (s), and time immobile (s). All testing and analysis were done blinded toward mouse experimental group, and experimental groups were randomized across trial times.

### Rotarod.

Mice were placed on a continuously rotating accelerating rotarod for mice (model 7650, Ugo-Basile). Once mice were secure on the rod at the lowest spin speed, the rotarod was switched to acceleration mode. The time from the beginning of the acceleration until the mouse fell from the rotating beam was recorded. Mice that exceeded the 10-minute time limit without falling were manually removed from the beam, and their times were excluded from the cohort. Three times were obtained for each mouse. Mice were given at least a full minute of rest time in a holding cage in between each trial. The experimenter was blinded toward mouse experimental group, and experimental groups were randomized across trial times.

### Wire walk.

Mice were placed on a 58 cm long, 1 cm circumference wire, held 49 cm high between 2 wooden poles, affixed to a wooden base. Once the mouse had gripped the wire with both front and rear paws they were released and allowed to move freely along the wire. Mice were given 3 trials with 1- to 3-minute-long rest periods in between trials. Video recordings were used to obtain exact measurements for time and distance travelled. All testing and analysis were done blinded toward mouse experimental group, and experimental groups were randomized across trial times.

### Intracisternal CSF tracer infusion.

These experiments were done as described previously (6, 81, 82). Fluorescent CSF tracer (bovine serum albumin, Alexa Fluor 647 conjugate, 66 kDa; Invitrogen, Life Technologies) was formulated in artificial CSF at a concentration of 0.5% weight by volume. Anesthetized mice were fixed in a stereotaxic frame, the CM surgically exposed, and a 30-gauge needle was connected to PE10 tubing filled with the tracer was inserted into the CM. Ten microliters of CSF tracer was infused at a rate of 2 microliters per minute for 5 minutes with a syringe pump (Harvard Apparatus). Animals were sacrificed by decapitation, and the brain was removed 30 minutes after the start of intracisternal infusion. The brain was fixed overnight in 4% paraformaldehyde in PBS (Sigma-Aldrich).

Coronal vibratome slices (100 μm) were cut and mounted. For all experiments except the time-restricted feeding experiments in healthy animals, tracer influx into the brain was imaged ex vivo by macroscopic whole-brain and whole-slice conventional fluorescence microscopy (Olympus, Stereo Investigator Software). For the time-restricted feeding experiments in healthy animals, slices were imaged on an Olympus BX63 microscope (Olympus, Stereo Investigator Software). Experiments performed with high-dose melatonin were imaged using an Olympus BX63 microscope, while experiments performed with low-dose melatonin were imaged using Olympus MVX10.

Tracer influx was quantified by a blinded investigator using ImageJ software (NIH). Each slice was manually outlined, and the mean fluorescence intensity within the outline was measured. An average fluorescence intensity was calculated between 6 slices for a single animal, resulting in a single biological replicate. Equivalent slices were used for all biological replicates (AP from bregma +1.2 to –1.8 mm with 500 �m intervals). Representative images were chosen based on being within 1 SD of the group mean and reimaged on the Olympus BX63 microscope (Olympus, Stereo Investigator Software), and brightness was adjusted equally across all experimental groups to allow accurate comparison of tracer distribution. These were then cropped around the outline of the coronal section and put in series for better visualization of tracer distribution along the anterior-posterior axis.

For acute KL001 experiments, melatonin experiments, and light experiments, brains were harvested a half-hour after the start of glymphatic influx assessment (for example, KL001 administered at ZT4 would have glymphatic influx at ZT5, and brains would have been collected at ZT5.5). For glymphatic influx after 8 days of timed feeding, 2 weeks of melatonin, 24 hours after photothrombotic stroke, and 8 day KL001, all experiments were done around midday (ZT5–ZT7).

### Immunohistochemistry and stroke quantification.

Equivalent 100 μm thick brain sections with visible stroke tissue, in a region from −0.1 mm to −3 mm from bregma were selected from each mouse. Slices were permeabilized with 0.1% Triton X-100 in PBS, blocked with 10% normal donkey serum (Jackson Immunoresearch) in PBS with 0.03% Triton X-100 for 1 hour and incubated with primary antibody overnight, followed by 3 washes in PBS and incubation with the fluorophore-linked secondary antibodies (Invitrogen, Life Technologies) for 1 hour. Stained slices were mounted with ProLong Gold antifade reagent (Invitrogen, Life Technologies). Primary antibody used were mouse anti-GFAP (1:500 dilution, Millipore, lot no. MAB360) and rabbit anti-MAP2 (1:1,000 dilution, Millipore, lot no. AB5622). Secondary antibody used were Cy2 donkey anti-mouse (1:500 dilution, Jackson Immuno Research, lot no. 156122, code 715-225-151) and Cy3 donkey anti-rabbit (1:1,000 dilution, Jackson Immuno Research, lot no. 155995, code 711-165-152). Cell nuclei were identified using DAPI (1:5,000 dilution, Invitrogen, lot no. D1306). Images were taken by a separate investigator who randomized file names, and analyses were done with investigators blinded to experimental group. Stroke regions were defined by visibly greater GFAP fluorescence and reduced MAP2 fluorescence accompanied by altered cellular morphology or complete cell death/absence. Total volume was estimated by measuring the area of affected tissue on each slice, multiplying by the 100 μm thickness of the slices to obtain a volume estimate of stroke tissue from each slice, and taking the sum of all slice volume estimates.

### Cytokine assay.

Brain and blood from wild-type (C57BL/6) mice were harvested at ZT6. Mice were anesthetized with KX, and the vena cava was exposed. 0.1 mL heparin (1:5 dilution, Fresenius Kabi) in PBS was injected into the vena cava to prevent clotting. Then, blood was extracted through vena cava, and the animal was perfused with ice-cold PBS. The brains were removed from the mice. Blood samples clotted for 30 minutes under room temperature and then centrifuged at 1,000 RCF, 4°C for 10 minutes. Brains were cut in half along the sagittal plane with the cerebellum removed, and one half was used for protein extraction. Brain was homogenized in lysis buffer (25 mM Tris•HCl pH 7.6, 150 mM NaCl, 1% NP-40, 1% sodium deoxycholate, 0.1% sodium dodecyl sulphate) with a protease inhibitor cocktail (1:100 ratio, Sigma-Aldrich) using a Fisherbrand 150 homogenizer. Samples were kept at 4°C or on ice to avoid freeze thaw cycles. Tissue samples were sonicated 3 times for 10 seconds (Branson Sonifier 150D, Emerson) and centrifuged at 13,000*g* for 20 minutes at 4°C. Supernatant was transferred to a new tube, and brains were diluted 1:10 in lysis buffer. Protein concentration was determined by a Pierce BCA assay (Thermo Fisher). All samples were shipped to Eve Technology Company for the Mouse High Sensitivity 18-Plex Discovery Assay. To compare total cytokine burden, cytokine levels were normalized to the highest value across all mice. For example, there were 13 mice total in the vehicle- and KL001-treated experiment in Figure 5C. To get the normalized value of IFN-γ, all values would be normalized to the highest of those 13 mice. This was repeated for each individual cytokine. Raw values for each cytokine as reported by EVE technology are shown in Supplemental Figure 5.

### MRI data acquisition processing.

MRI experiments were approved by and conducted in accordance with guidelines from the University of Rochester Committee in Animal Resources. Mouse brain anatomical imaging was performed as described in our earlier studies (83, 84). MRI data were acquired using a 9.4 T Biospec (Bruker) small-animal system equipped with 740 mT/m gradients and a cryogenically cooled ^1^H surface CryoProbe (Bruker). Animals were briefly exposed to isoflurane (2%–3% vol.) for placement into the restrainer and positioned into the magnet with a laser-controlled system for the animal cradles. Respiratory frequency and body temperature (maintained with a water heating pad) were monitored throughout the experiment using a small-animal monitoring system (SAI Inc.). High-resolution morphological images were acquired using T_2_-weighted rapid acquisition with relaxation enhancement (RARE) (85) imaging sequence (echo time/repetition time [TE/TR] = 33/2,500 ms, 2 averages at RARE factor of 8). Mouse whole brain was covered using 25 slices (0.5 mm slice thickness) at planar spatial resolution of 78 × 78 μm^2^ with a field of view of 20 × 20 mm^2^ and an acquisition matrix of 256 × 256. Stroke lesions were manually segmented using AMIRA (Thermo Fisher Scientific) (86), and the lesion volume was computed using a custom Matlab script.

### Statistics.

Statistical analyses were performed in GraphPad Prism version 7.0. For comparisons of means in samples with normal distributions and homogeneous variances, an unpaired 2-tailed *t* test was used for 2 groups. For more than 2 groups, an ANOVA was used for comparisons between means, followed by Tukey’s honestly significant difference (HSD) post hoc comparisons. In cases of a nonnormal distribution or unequal variances, a nonparametric Mann-Whitney test or an unpaired 2-tailed *t* test with Welch’s correction was used for comparisons between 2 means, and a nonparametric Kruskal-Wallis test was used for comparisons between more than 2 means, followed by Dunn’s multiple comparisons test. In the case of longitudinal time points, data were analyzed using a repeated-measures 2-way ANOVA. All experiments were 2-tailed. Significance was defined as *P* < 0.05. For exact test statistics, see Supplemental Tables 1 and 2.

### Study approval.

All experiments were approved by the University of Rochester Medical Center Committee on Animal Resources.

### Data and materials availability.

All data are available in the main text, supplemental materials, and the Supporting Data Values file.

## Author contributions

Conceptualization: MN and LMH. Methodology: EW, YZ, AC, WS, TMA, and LMH. Investigation: EW, YZ, AC, VSV, EN, MJG, ED, EB, WS, TMA, YM, and LMH. Visualization: EW, YZ, ED, and LMH. Funding acquisition: MN and LMH. Project administration: MN and LMH. Supervision: MN and LMH. Writing — original draft: EW, YZ, and LMH. Writing — review and editing: EW, YZ, AC, VSV, EN, MJG, ED, EB, WS, TMA, YM, MN, and LMH. EW and YZ are co–first authors as they collected the data for every figure of the manuscript and contributed much to the writing. EW is listed first for the amount of time spent working the project.

## Conflict of interest

The authors have declared that no conflict of interest exists.

## Funding support

This work is the result of NIH funding, in whole or in part, and is subject to the NIH Public Access Policy. Through acceptance of this federal funding, the NIH has been given a right to make the work publicly available in PubMed Central.

- American Heart Association Career Development Award 941177 to LMH.
- American Heart Association 26BCDA1622757 to LMH.
- Lundbeck Foundation grant R386-2021-165.
- Novo Nordisk Foundation grant NNF20OC0066419.
- NIH grants U19NS128613, R01AT012312, R01AT012707, R01AT011439.
- Dr. Miriam and Sheldon G. Adelson Medical Research Foundation.
- Simons Foundation grant 811237.
- Cure Alzheimer Fund, BEE Consortium, to MN.

## Supplementary Material

Supplemental data

Supplemental table 1

Supplemental table 2

Supporting data values

## Version 1 06/15/2026

Electronic publication
