# Supplementary material for: Chronotherapy to reinforce circadian rhythms improves poststroke outcomes and glymphatic function in mice
Source: J Clin Invest. 2026 Jun 15;136(12):e201800. doi: 10.1172/JCI201800 (PMC13262723; doi:10.1172/JCI201800)
Supplement: Supplemental data [file jci-136-201800-s130.pdf]

Supplemental Figures:

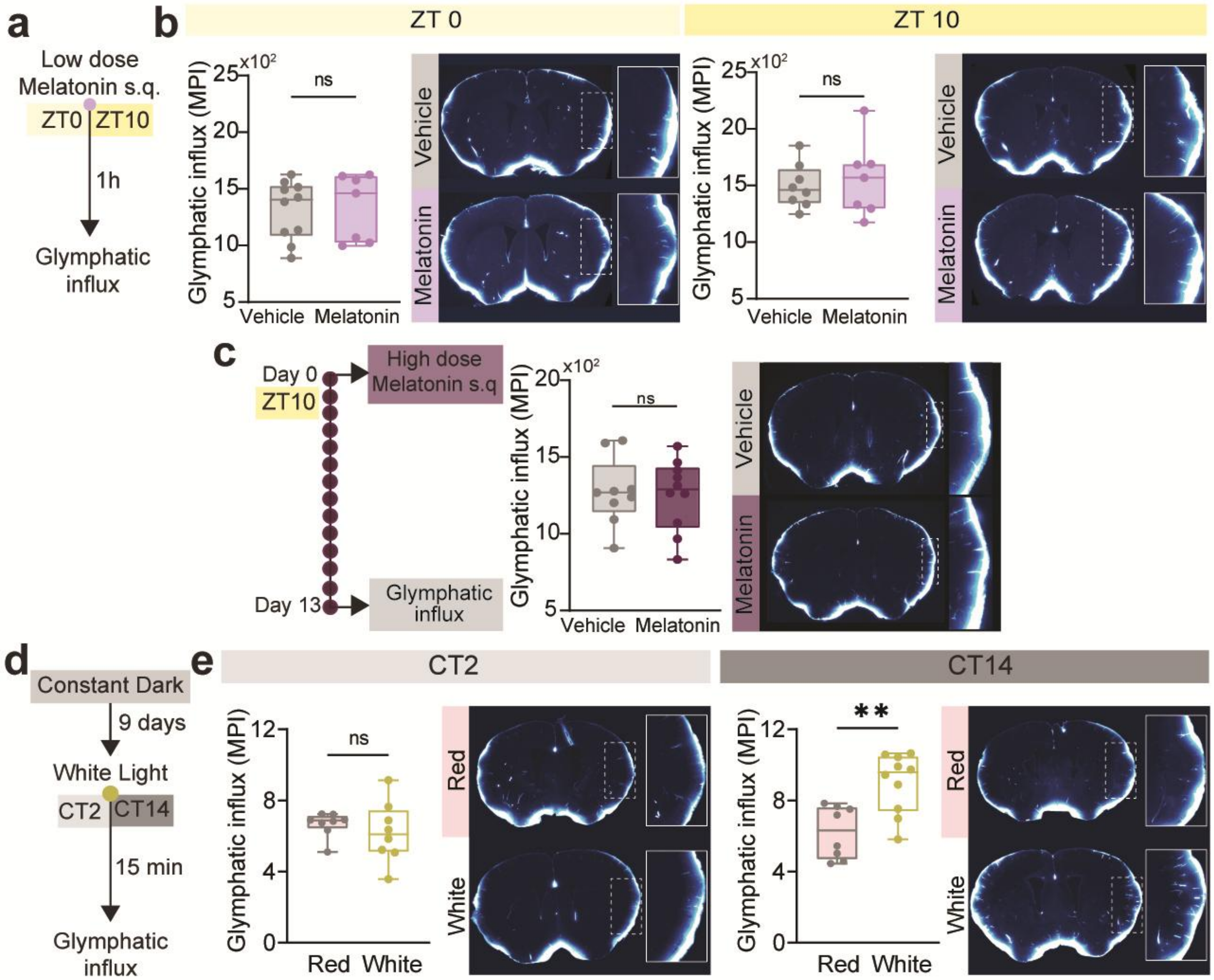

Fig. S1. Circadian interventions increase glymphatic influx. a, Experimental design for low dose sub-cuetaneus (s.q.) melatonin administration. **b,** Boxplots of mean pixel intensity (MPI) of glympahtic influx and representative coronal section for experiments done at Zeitgeber Time 0 and 10 (ZT0 and ZT10, lights on at ZT0). **c**, Experimental design for two-week treatment of high dose subcuetaneous (s.q.) melatonin administration (left), boxplots of mean pixel intensity (MPI) of glymphatic experiments (middle) and represetnative coronal sections (right). **d**, Experimental design for acute white light pulses in constant dark. Light pulses were given at Circadian Time 2 (CT2, 2 hours after activity offset) or CT14. **e**, Same as (**b**) but for the white light administration. For all boxplots: minima is minimum value, maxima is maximum value, center is median, and quartiles shown by box and whiskers with individual mice shown

as colored dots. ns: not significant, *p<0.05, **p<0.01. Inset areas are marked with a dotted line on coronal sections and 2x magnified. Statistical comparisons were either an unpaired t-test, an unpaired t-test with Welch's correction, or a Mann-Whitney test. All statistics are in Table S2.

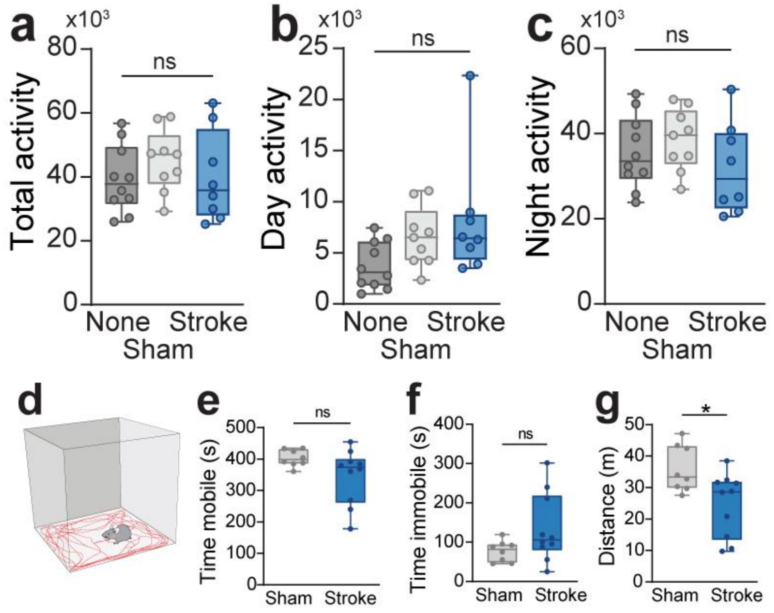

Fig. S2. Behavioral assessment of mice post photothrombotic stroke. Boxplot of total wheel running activity across 24h (**a**), wheel running during the light phase (**b**), and wheel running in the dark phase (**c**) in mice that received no surgery (None, dark gray), sham surgery (light gray), or photothrombotic stroke (blue). (**d**) Mice were placed in an open field. Box1plots of time mobile (**e**), time immobile (**f**), and total distance traveled (**g**) for mice with sham surgery (gray) or photothrombotic stroke (blue). For all boxplots: Minima is minimum value, maxima is maximum value, center is median, and quartiles shown by box and whiskers with individual mice shown as colored dots. ns: not significant, *p<0.05. **a-c:** one-way ANOVA, **e-g:** unpaired t-test, or with an unpaired t-test with Welch's correction. All statistics in Table S2.

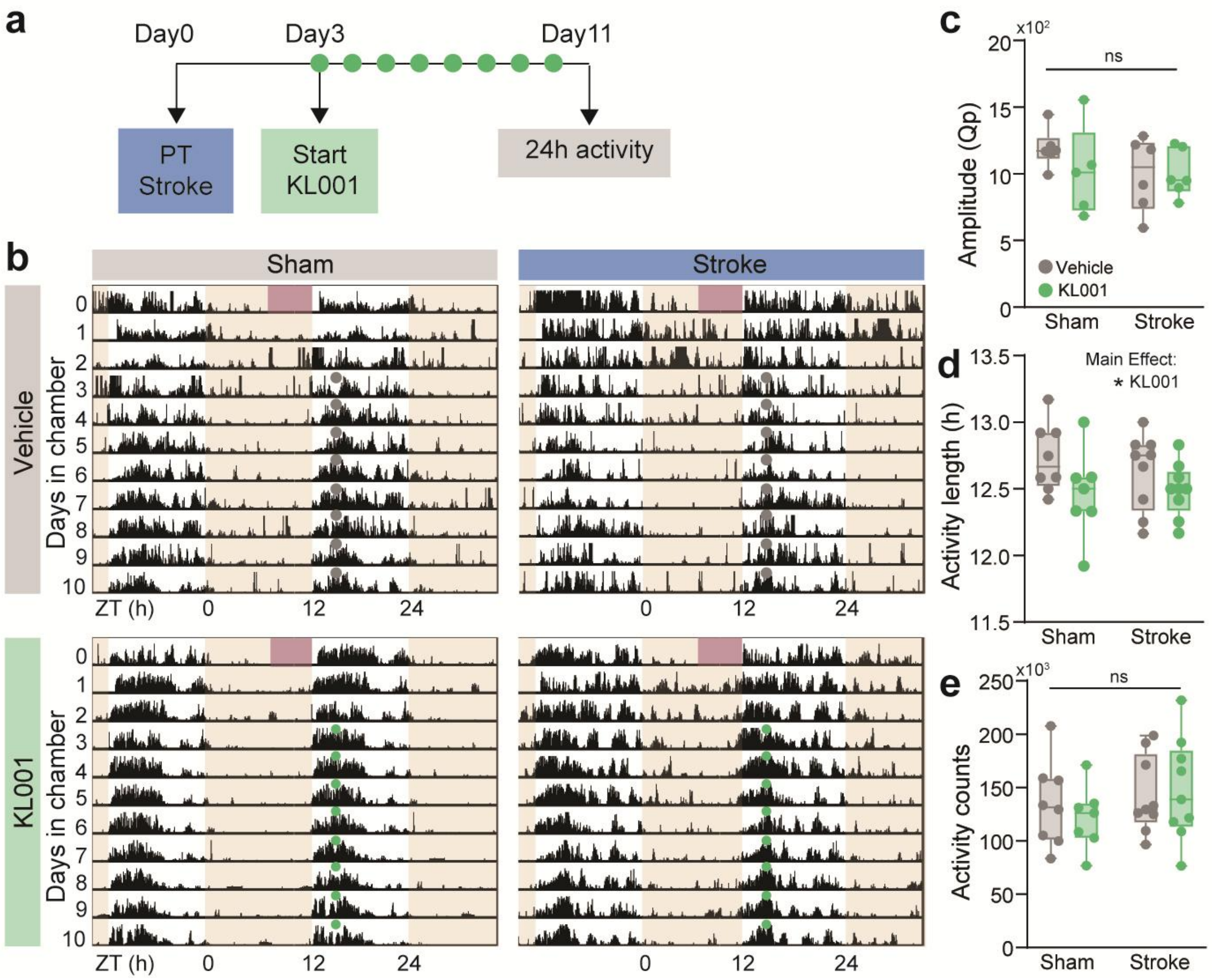

Fig. S3. KL001 after photothrombotic stroke shortens activity length. a, Experimental design. **b**, Representative double-plotted actograms of general cage activity for mice in experiment **a**. Tan, lights on; white, lights off; black tick marks, general cage activity; purple block, time of stroke or surgery. Colored dots, time of injection of vehicle (gray) or KL001 (green). Boxplots of chi-squared periodogram calculated rhythmicity amplitude at 24h (**c**), activity length (**d**) and daily activity counts (Boxplot of chi-squared periodogram calculated rhythmicity amplitude at 24h (**e**). For all boxplots: Minima is minimum value, maxima is maximum value, center is median, and quartiles shown by box and whiskers with individual mice shown as colored dots. ns: not significant, *p<0.05, **p<0.01. In **c**, **d**, **e**, individual dots are single cages of double-housed mice. **c-e:** Two-way ANOVA tests. All statistics in Table S2.

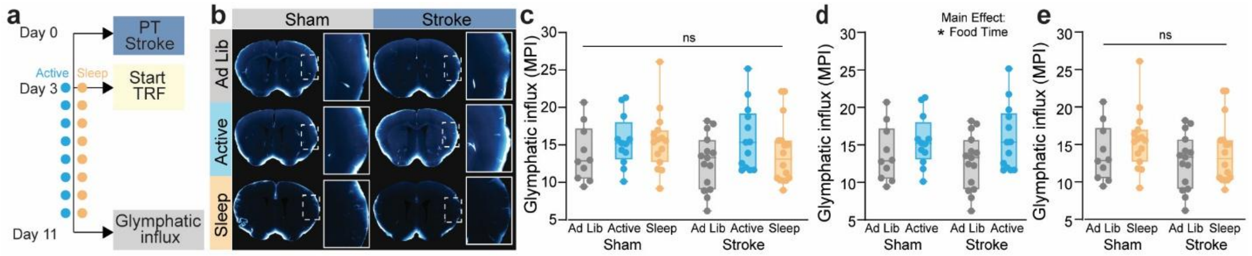

Fig. S4. Active-phase restricted feeding improves glymphatic influx after photothrombotic stroke. a, Experimental design. **b**, Representative coronal brain sections for glymphatic influx analysis (left). Dotted yellow box indicates 250x expansion inset on the right. **c-e**, Boxplots of glymphatic influx measured in mean pixel intensity (MPI). For all boxplots: Ad Lib, gray; Active, active phase restricted feeding, blue; Sleep, sleep phase restricted feeding, orange. Minima is minimum value, maxima is maximum value, center is median and quartiles shown by box and whiskers, with individual mice shown as colored dots, ns: not significant, *p<0.05. **c-**e: Two-way ANOVA tests. All statistics in Table S2.

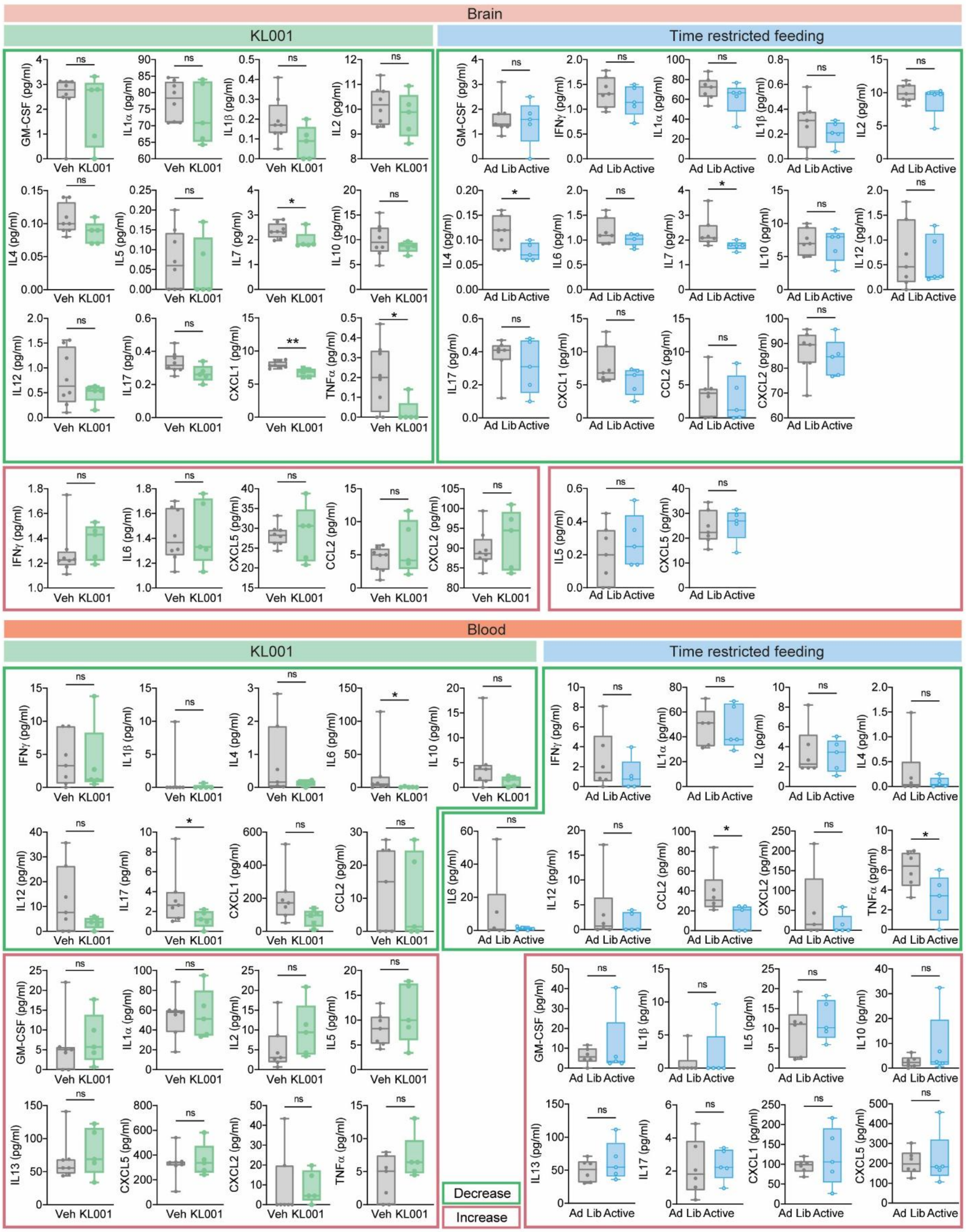

Fig. S5. Individual tissue-specific cytokine comparisons with chronotherapy. Boxplots of all brain (tan) and blood (red) cytokines with and without KL001 (green) or active phase time restricted feeding

(Active, blue) chronotherapy after stroke. Green boxes indicate a decreased mean group value with chronotherapy compared to control, red an increased mean group value. For all boxplots: Minima is minimum value, maxima is maximum value, center is median, and quartiles shown by box and whiskers with individual mice shown as colored dots. ns: not significant, *p<0.05, **p<0.01. All statistics in Table S2."maxima is maximum value, center is median, and quartiles shown by box and whiskers with individual mice shown as colored dots. ns: not significant, *$p<0.05$, **$p<0.01$. Comparisons were made with either an unpaired t-test, an unpaired t-test with Welch's correction, or a Mann-Whitney test. All statistics in Table S2.

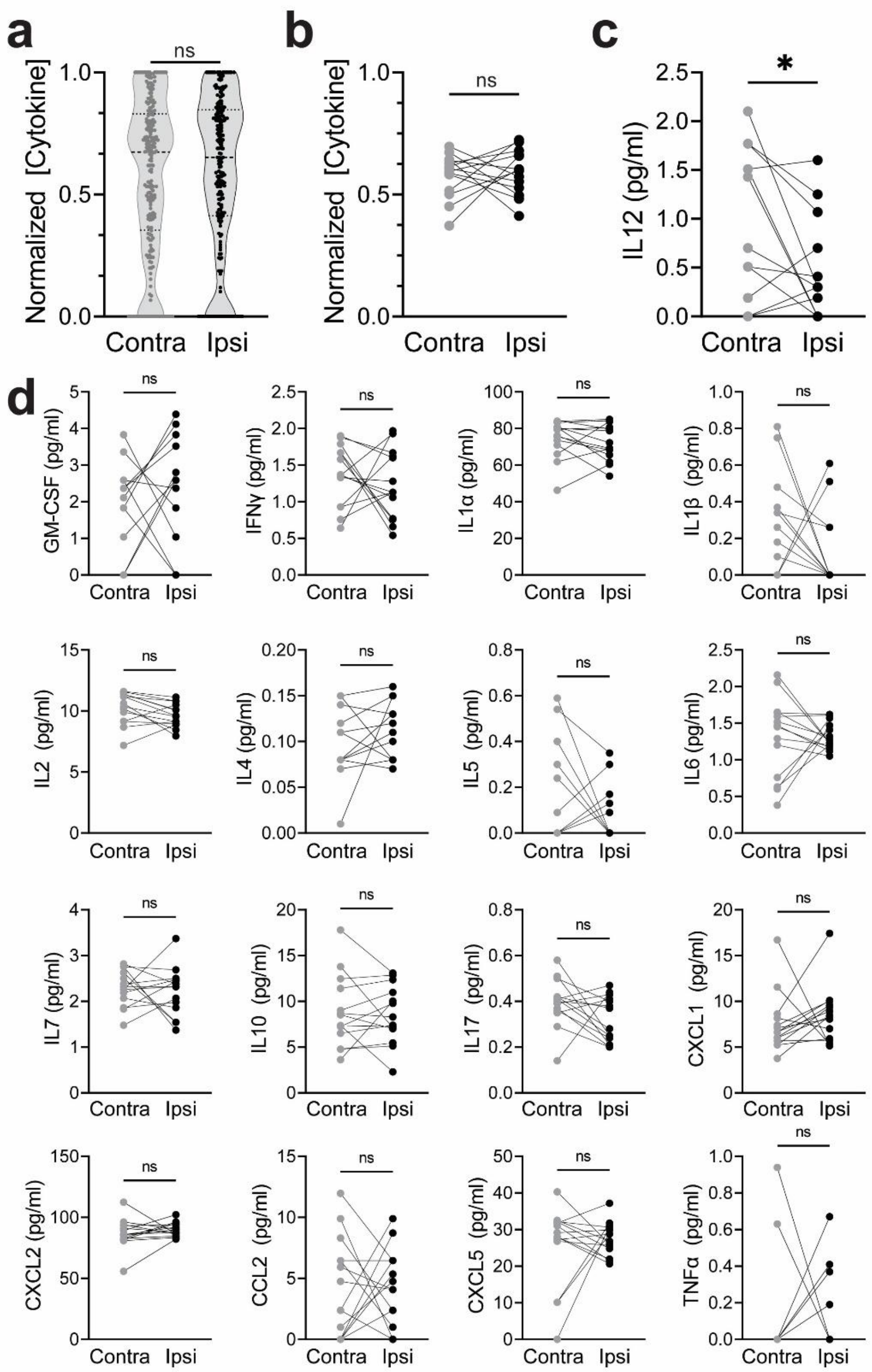

Fig. S6. Brain cytokine concentrations are not different contra- or ipsi-lateral to 11 days old photothrombotic stroke. a, Violin plot of all individual brain cytokine concentrations normalized to the peak value on the contralateral (contra, gray) and ipsilateral (ipsi, black) side of the brain after photothrombosis. Width reflects concentration of data, dotted lines are upper and lower

quartile limits, solid black line indicates median, individual points are individual cytokine values. **b**, Scatterplot of normalized cytokine concentration by mouse. **c**, Scatterplot of IL12 concentration 11 days after stroke. **d**, Scatterplots of individual cytokines between contra- and ipsilateral brain sides 11 days after photothromobosis. For all scatterplots: Solid connected lines indicate paired values from a single brain, individual mice shown as colored dots, ns: not significant, *$p<0.05$. All comparisons were either a paired t-test or a Wilcoxon test. All statistics are in Table S2.
